# Identification and Estimation of Solute Storage and Release in Karst Water Systems, South China

**DOI:** 10.3390/ijerph17197219

**Published:** 2020-10-02

**Authors:** Liang Zhang, Mingming Luo, Zhihua Chen

**Affiliations:** 1 School of Environmental Studies, China University of Geosciences, Wuhan 430074, China; zhang-liang@cug.edu.cn (L.Z.); zhchen@cug.edu.cn (Z.C.); 2 Key Laboratory of Karst Dynamics, MNR/GZAR, Institute of Karst Geology, Chinese Academy of Geological Sciences, Guilin 541004, China

**Keywords:** solute transport, storage and release, tracer test, karst water system, concentrated recharge

## Abstract

Solute storage and release in groundwater are key processes in solute transport for groundwater remediation and protection. In karst areas where concentrated recharge conditions exist, pollution incidents can easily occur in springs that are hydraulically connected to densely inhabited karst depressions. The intrinsic heterogeneity common in karst media makes modeling solute transport very difficult with great uncertainty. Meanwhile, it is noteworthy that solute storage and release within subsurface conduits and fissures exhibit strong controlling function on pollutant attenuation during underground floods. Consequently, in this paper, we identified and estimated the solute storage and release processes in karst water systems under concentrated recharge conditions. The methodology uses the advection–dispersion method and field tracer tests to characterize solute transport in different flow paths. Two solute transport pathways were established (i.e., linear pathway (direct transport through karst conduits) and dynamic pathway (flow through fissures)). Advection–dispersion equations were used to fit the breakthrough curves in conduit flow, while the volume of solute storage in fissures were calculated by segmenting the best fitting curves from the total breakthrough curves. The results show that, greater recharge flow or stronger dynamic conditions leads to lower solute storage rate, with the storage rate values less than 10% at high water level conditions. In addition, longer residence time was recorded for solute exchange between conduits and fissures at the low water level condition, thereby contributing to a higher solute storage rate of 26% in the dynamic pathway.

## 1. Introduction

In the karst area of South China, intense rainfall events frequently occur due to the subtropical monsoon climate, which generates overland flow that conveys sediments, solutes, or pollutants. These sediments and solutes often find their way into the aquifer system through sinkholes and other karst fractures. Consequently, there is a rapid solute transport based on these hydrological responses, underground floods, resulting in great changes in the physical and chemical properties of karst groundwater [1,2].

Karst depressions and sinkholes are well developed in South China, which are usually inhabited with large settlements and agricultural activities. The karst water systems usually comprise of multiple heterogeneous aquiferous media (i.e., caves, conduits, fissures, and matrix), which make karst aquifers extremely vulnerable to pollution [3]. The depressions are therefore highly susceptible to groundwater contamination due to this convergence of high anthropogenic activities and favorable meteorological and geomorphological characteristics, which produce autogenic recharge from overland flow. It is therefore significant to model and characterize contaminant transport under these hydrological and morphological conditions for groundwater remediation and protection. However, simulating contaminant transport in karst water systems has been a challenge [4]. The main difficulty in characterizing flow through the system is based on the heterogeneity of the aquifer matrix, which has multiple flow pathways that are defined by varying hydraulic and geometric properties. To adequately define the solute transport mechanism in such a matrix, the hydrodynamics under different recharge conditions have to properly modeled.

Solute storage and release are a very common phenomenon in the solute transport process, which has attracted more and more attention from researchers in recent years [5]. For example, in systems with surface water and groundwater interaction, riparian sediments store water and solutes when the river level rises, and the solutes stored in the riparian sediments release into the river again when the river level drops [6]. Similarly, sinkholes carry a large amount of overland flow and solutes into the karst aquifer under concentrated recharge conditions including different types of pollutants from the land surface, where the water and solute exhibit an interchange between karst conduits and fissures [7,8]. The solute storage and release processes occur in the flow and solute exchange between conduits and fissures under different hydrodynamic conditions. The characterization of solute storage and release processes is the key problem in the solute exchange between the conduit and fissure flow, which controls solute or pollutant transport and retention during underground floods.

In terms of the exchange, transient storage, and release processes during solute transport, previous studies were focused on solute transport, storage, adsorption-resolution, and biogeochemical processes in the river hyporheic zones [9,10]. The exchange flow between karst conduits and matrix have attracted the attention and been modeled by the Conduit Flow Process (CFP) code [11,12], but the exchange solute has rarely been discussed. Some studies have discussed the transient storage capacity of solutes or pollutants in the surface ditches [13]. As for the solute transport mechanism in karst water systems, the advection–dispersion process was mainly considered in previous studies [4]. For example, Cholet et al. (2017) used the reverse advection–dispersion method to compare the exchange of lateral flow and solute between karst conduits in the saturated zone and vadose zone within underground flooding [14]. Others have reported the effects of flow recessions and solution pool on solute transient storage through laboratory experiments [15,16], and the solute exchange in the sediments of karst conduits was also discussed [17]. However, less attention has been paid on the solute transient storage and release along the fast flow in karst conduit and fissure systems during underground floods.

Overall, the special hydrogeological structure of karst water systems has led to the extremely high vulnerability of groundwater around the world and in South China specifically. The sinkholes and karst conduits become the dominant channels transporting underground floods and pollutants generated by autogenic recharge. Characterizing the solute transport mechanism is the foundation for studying the self-purification mechanism for karst water, which provides scientific basis for pollution control and protection in underground rivers or springs. This paper chose a typical karst study area in South China, which is located in the Xiangxi River basin in western Hubei Province. This important agricultural area is facing high risk from non-point source pollution, especially in the karst area with abundant depressions and sinkholes. To our knowledge, the processes of solute storage and release in karst water systems have been rarely quantitatively recognized and simulated. This paper aimed to conceptualize different solute transport paths in karst water systems in South China, and estimate the solute storage and release through karst conduit and fissure systems.

## 2. Study Area Descriptions

**Figure 1:**
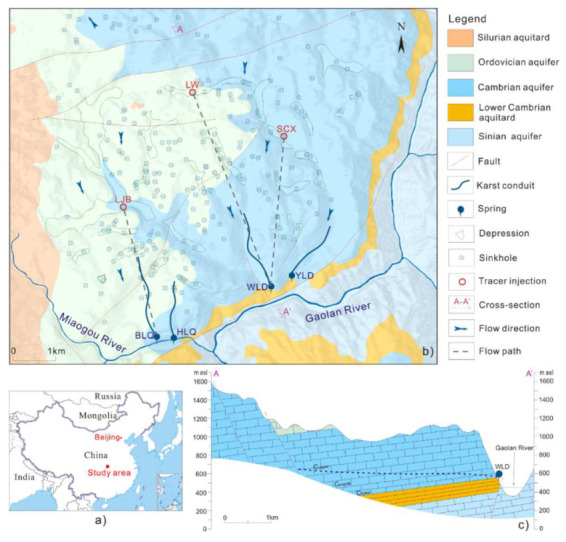
(**a**) Location of the study site in China. (**b**) Hydrogeological map of the study area, modified after Luo et al. (2016, 2018) [18,19]. (**c**) Schematic geological-hydrogeological cross section of WLD spring along the A–A’ line shown in (**b**), modified after Luo et al. (2016) [18].

The study area is situated in Xingshan County, western Hubei Province, South China (Figure 1a). This area is a typical karst trough valley in the Xiangxi River basin, a tributary of the Changjiang River. The region has a subtropical monsoon climate with four seasons and abundant rainfall, with an average annual rainfall of 900–1200 mm. Uplift and erosion have produced a steep terrane of middle mountains and deep ravines, which features complex karst landforms and great topographic relief. The northern parts form the recharge area of several springs, with elevations ranging from 900 to 1100 m above sea level (Figure 1b). The discharge area is in the south, at the northern bank of Gaolan River and Miaogou River (350 to 550 m above sea level (a.s.l.)). The land use of the karst depressions are mainly settlements and agricultural land, and there is no industry in the study area. Agricultural non-point source pollution and domestic pollution are the main risks for karst groundwater.

Thick units of carbonate rocks (limestone and dolostone) are widespread in the study area, mainly in the Late Precambrian, Cambrian, and Ordovician, which form the main karst aquifers (Figure 1b). Mudstone and shale of the Lower Cambrian and Silurian are present as the regional aquiclude in this area. The main karst aquifer of Ordovician limestone, Lower Cambrian limestone, and Middle to Upper Cambrian dolostone is all interconnected, which lies on top of the regional aquiclude of Lower Cambrian mudstone and shale (Figure 1c). Overall, it is a well-developed karst environment with several karst features such as depressions with numerous sinkholes, dolines, and springs. There is no allogenic recharge source in this area, and rainfall is the main recharge source of groundwater, which easily percolates to the aquifers through sinkholes such as Liujiaba (LJB), Longwan (LW), and Shicaoxi (SCX) sinkholes. The sinks receive overland flow, which forms an autogenic recharge generated from around the karst depressions.

Hydrogeological investigations [18] have established the dominant groundwater flow mechanism to be through subsurface conduits and fissures which ultimately discharges through springs. There are four major perennial springs in the study area: Yunlongdong (YLD), Wulongdong (WLD), Heilongquan (HLQ), and Bailongquan (BLQ), which are situated along the north bank of the Gaolan River and Miaogou River (Figure 1b). YLD, WLD, and HLQ springs emerge near the base of the Cambrian karst aquifer, where downward flow is obstructed by the Lower Cambrian aquitard (Figure 1c), and the geological settings at the outlets of these springs are similar. The BLQ spring emerges from the overlying Upper Cambrian karst aquifer. These springs are with average discharge rate ranging from 0.04 to 0.15 m^3^/s, while the maximum flow can be up to several cubic meters per second after rainstorms [18].

## 3. Materials and Methods

### 3.1. Tracer Tests and Fitting Method for Breakthrough Curves

Artificial tracer tests are often used to determine the recharge areas of karst springs and calculate groundwater velocity and some other hydrogeological parameters. Furthermore, they are used to quantitatively characterize and simulate groundwater flow and solute transport processes, or evaluate groundwater vulnerability, etc. [20,21,22,23,24,25]. An artificial tracer test in karst areas not only recognizes the recharge source of karst groundwater, but also identifies the spatial and structural characteristics of a karst system [18,26,27]. These applications have become essential to investigating karst hydrodynamic characteristics and solute transport mechanisms.

In this study, recharge sources, flow paths, and drainage structures were defined by artificial tracer tests conducted in 2013 and 2014 during different periods of rainfall intensity. Fluorescent dyes of uranine and rhodamine were used as solute tracers, which are reasonably conservative, safe, stable in the environment, and do not interact with the aquifer. The concentrations of uranine and rhodamine were measured by a fluorometer (GGUN-FL Fluorometer) at the downstream of four major springs. At a low water level, rhodamine was injected in LJB, a sinkhole located in a karst depression (Figure 1b), with perennial inflow that averages about 5 L/s during summer. Similarly, at high water level, tracers were injected into LW and SCX sinkholes where rainstorms generate the inflow of overland flow (Figure 1b).

Five groups of artificial tracer tests were conducted in the study area to determine the flow paths, and their tracer breakthrough curves provided the basic data for our analyses. Tracer test along LJB–BLQ was conducted under a low water level condition, while tracer tests along LW and SCX to WLD were conducted under high water level conditions with different rainfall intensity. More details about the tracer tests can be found in Luo et al. (2016) [18], who also elucidates the recharge and drainage structure of the study area.

During turbulent flow in karst conduits, mechanical dispersion is most important in the flow direction, and longitudinal advection–dispersion theory describes the displacement of substances with a one-dimensional fluid () [28]. An analytical solution () can be applied to a range of initial and boundary conditions [29]. This solution assumes that the tracer mass is instantaneously injected into a homogeneous and isotropic medium, and the fluid flows at a steady velocity with constant dispersion coefficient and fluid density. However, karst aquifers are difficult to model because as aquifers they are heterogeneous and anisotropic, and as conduit networks, they have unsteady open and closed channels of varying scale. Nevertheless, it is still worthwhile properly conceptualizing the structure of a karst water system. Here, we are trying to find the best-fit approach to better interpret the tracer test results and determine the transport parameters.

(1) $$\frac{\partial C}{\partial t}=D_{L}\frac{\partial^{2}C}{\partial x^{2}}-v\frac{\partial C}{\partial x}$$

(2) $$C(x,t)\;=\;\frac{M}{A\sqrt{{4\pi D}_{L}t}}exp\left[\frac{{-(x-vt)}^{2}}{{4D}_{L}t}\right]$$

where *C* is the tracer concentration; *M* is the tracer mass; A is the cross-sectional area determined by discharge volume and longitudinal distance; *D_L_* is the longitudinal dispersion coefficient; *x* is the longitudinal distance; *t* is the time after tracer injection; and *v* is the effective flow velocity.

### 3.2. Conceptual Model

The typical karst water system in South China often features an extensively developed network of subsurface karst conduits and fissures, with abundant sinkholes in karst depressions at the surface. Fast underground flow that rapidly responds to rain events always occurs in these mountainous and well karstified areas. Under concentrated recharge conditions, the karst water systems mainly get inflows from sinkholes, which results in rapid hydrological responses at the outlet of karst springs.

**Figure 2:**
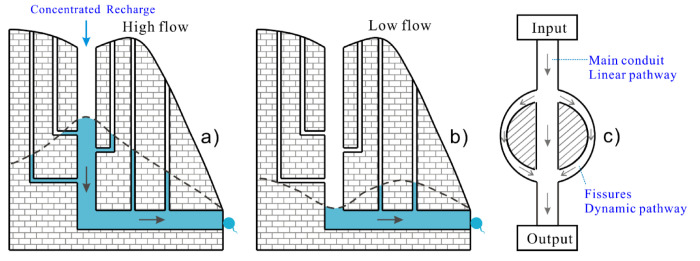
Conceptual sketch of the two solute transport paths in the karst conduit-fissure system under concentrated recharge condition. (**a**) Water level in the conduit is higher than that in the fissure, that conduit flow infiltrates into the fissure flow. (**b**) Water level in the conduit is lower than that in the fissure, that conduit flow gets recharge from fissure flow. (**c**) Two conceptual solute transport paths that shows longer flow path through the fissures.

Figure 2 describes a typical karst media with an interactive matrix between conduits and fissures. Conduit flows have much higher velocity due to associated geometrical and hydraulic head conditions such as conduit size and slope. Accordingly, when the conduit is full, the flow percolates into the fissures (Figure 2a), which flows back when the volume in the conduit decreases (Figure 2b). This interface between the conduit and fissures creates a dynamic flow that transmits and stores solutes in the fissures when the conduit is full and releases some of it back into the conduit under low flow conditions (Figure 2c).

The dynamic interface between the conduit and fissures define two transport and storage pathways, hereafter referred to as linear and dynamic pathways (Figure 2c). In the linear pathway, solutes always remain in the karst conduit transported by direct flow from the recharge inlet (sink) to discharge outlet (spring). For the dynamic pathway, solutes percolate into the fissures as bypass flow from the main conduit and under low flow conditions move back into the conduit before finally discharging from the outlet (spring) (Figure 2c). Consequently, the solutes undergo a more dynamic interface with the aquifer matrix and dominant flow pathway, which include storage and transport. This phenomenon also means a longer travel and residence time for the solute within the karst water system.

Single tracer injections can produce multiple-peak breakthrough curves in the cases of different flow paths developed in the karst aquifer. The multi-dispersion model assumes that these flow paths split directly at the injection point (sinkhole) and reunify at the outlet of spring. Each flow path is characterized by an individual advective flow velocity and dispersion. At the sampling point, the individual breakthrough curves superimpose to form a multi-peak curve at the spring outlet. Additionally, the decomposition of a multi-peak curve makes it possible to determine the individual flow velocities and dispersion of the individual flow paths [28]. The advection–dispersion equation (ADE) can be used to characterize the solute transport in different flow paths based on the sum and superposition of the solute transport process in different flow paths form the total breakthrough curve at the outlet of spring. The solute storage mainly occurs in the small and narrow flow paths (e.g., fissures), which still can get an individual breakthrough curve by segmenting the total breakthrough curve. By integrating the breakthrough curves in different transport paths, the transit amounts of solutes in different transport paths can be estimated.

## 4. Results and Discussion

### 4.1. Tracer Tests

**Figure 3:**
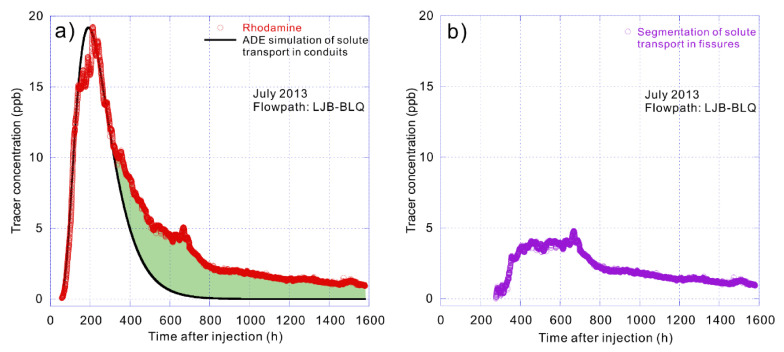
(**a**) Breakthrough curve of LJB–BLQ (Liujiaba-Bailongquan) and the fitting curve of solute transport in conduit flow using advection–dispersion equation (ADE). (**b**) Segmentation of solute transport in fissure flow that refers to the solute storage and release in the dynamic pathway.

On 5 July 2013, rhodamine was detected at BLQ spring 57.31 h after its injection at the LJB sinkhole 3.2 km away, with a maximum concentration of 19.25 ppb (part per billion) recorded 212.67 h after injection (Figure 3a). No effective rain fell in the two months following injection, and groundwater level remained low. The groundwater flow conditions represent the slow flow at low water level with low mean groundwater velocity of 15.18 m/h.

**Figure 4:**
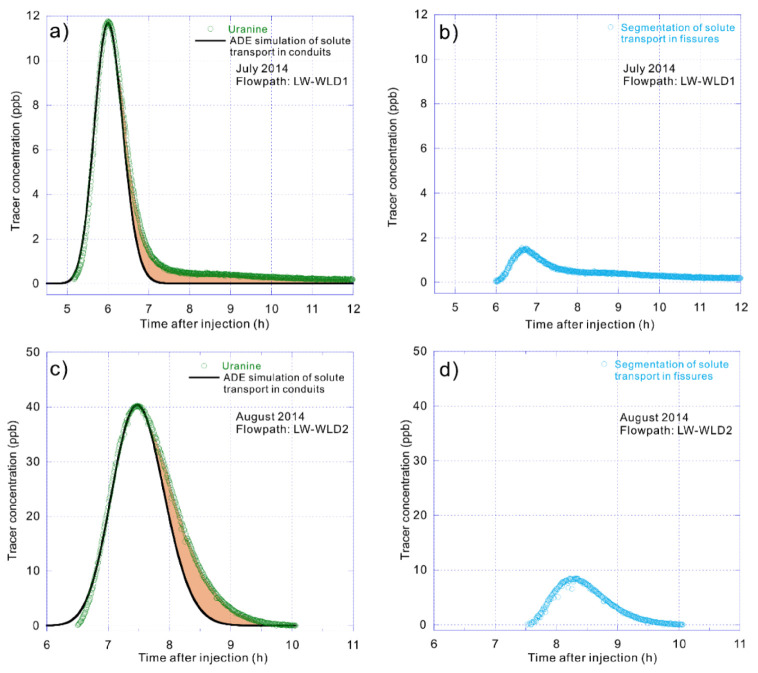
Breakthrough curves, fitting curves of solute transport in conduit flow and segmentations of solute transport in fissure flow of LW–WLD (Longwan-Wulongdong)**.** (**a**) Breakthrough curve of LW–WLD and the fitting curve of solute transport in conduit flow using advection–dispersion equation on 12 July 2014. (**b**) Segmentation of solute transport in fissure flow that refers to the solute storage and release in the dynamic pathway of LW–WLD on 12 July 2014. (**c**) Breakthrough curve of LW–WLD and the fitting curve of solute transport in conduit flow using advection–dispersion equation on 12 August 2014. (**d**) Segmentation of solute transport in fissure flow that refers to the solute storage and release in the dynamic pathway of LW–WLD on 12 August 2014.

**Figure 5:**
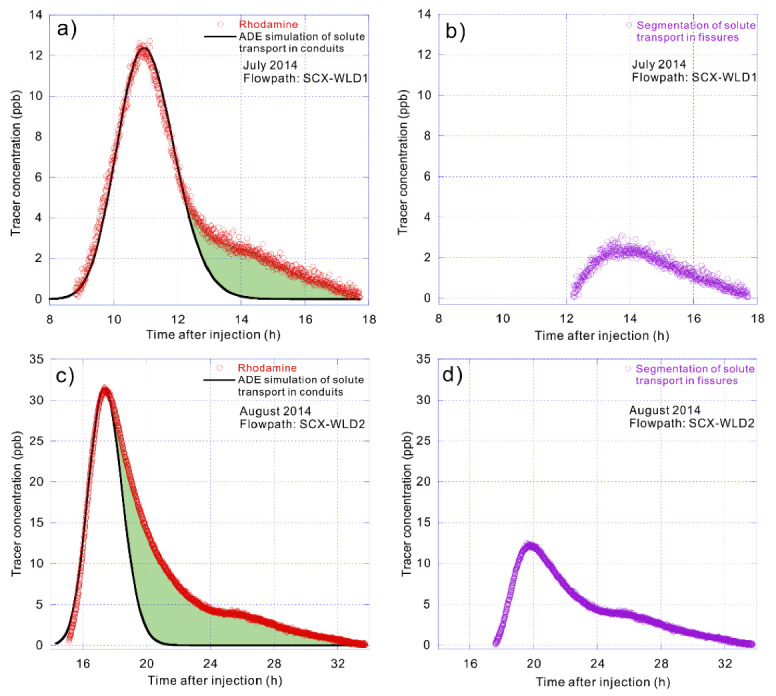
Breakthrough curves, fitting curves of solute transport in conduit flow and segmentations of solute transport in fissure flow of SCX–WLD (Shicaoxi-Wulongdong). (**a**) Breakthrough curve of SCX–WLD and the fitting curve of solute transport in conduit flow using advection–dispersion equation on 12 July 2014. (**b**) Segmentation of solute transport in fissure flow that refers to the solute storage and release in the dynamic pathway of SCX–WLD on 12 July 2014. (**c**) Breakthrough curve of SCX–WLD and the fitting curve of solute transport in conduit flow using advection–dispersion equation on 12 August 2014. (**d**) Segmentation of solute transport in fissure flow that refers to the solute storage and release in the dynamic pathway of SCX–WLD on 12 August 2014.

On 12 July 2014, uranine was detected at WLD spring only 5.17 h after its injection at the LW sinkhole more than 5 km away. It had a maximum tracer concentration of 11.77 ppb recorded after only about one additional hour (Figure 4a). Then, rhodamine was detected at WLD spring only 8.80 h after injection at the SCX sinkhole 3.6 km away, with the peak concentration of 12.72 ppb measured at WLD spring 11.13 h after injection (Figure 5a). The maximum groundwater velocities recorded for these two systems were 976 and 413 m/h, respectively.

Another group of tracer tests were conducted at the same injection and receiving points a month later, designated as WLD2 to distinguish this group from WLD1, which was conducted on 12 July 2014 (Table 1). On 12 August 2014, uranine injected at the LW sinkhole was detected at WLD spring 6.50 h later with a maximum concentration of 40.24 ppb recorded 7.47 h after injection (Figure 4c). Additionally, rhodamine that was injected at the SCX sinkhole was detected at WLD spring only 15.15 h later, with a peak concentration of 31.58 ppb attained at the WLD spring 17.38 h after injection (Figure 5c). The maximum flow velocities established on 12 August 2014 were somewhat slower, but still very rapid, being 776 and 240 m/h, respectively.

The presence of secondary and tertiary porosities in our study area creates varying degrees of flow types and conditions. In 2013, the flow path of LJB to BLQ was defined under low flow conditions with low volumes of water stored in the conduits due to the absence of autogenic recharge. Consequently, the fissures become a tertiary flow path and solute storage media. Groundwater velocity was found to be lesser than under high water level conditions as depicted by the long tail on the tracer breakthrough curve (Figure 3a). As for the tracer test conducted under intense precipitation in 2014, sinkholes generated inflow from the rapid overland flow, driving quick flows through conduits (main transport channels). Consequently, the durations of the breakthrough curves were short, showing a relatively symmetric single peak.

There was an increase in the rates of infiltration and conduit flow due to intense rainfall on 12 July, generating shorter transit times and larger dispersion, as presented in Table 1. The rapid transport rates of hundreds m/h defined by these tests establish the occurrence of large conduit flows that are typical in well-developed karst environments. The two groups of comparative tracer tests show that, under the concentrated recharge conditions, the actual velocity of the fast flow can reach up to hundreds m/h per hour, and it also conveys the solute more rapidly, resulting in a shorter duration of breakthrough curve. These curves and their tails tend to behave very differently due to associated flow pathway and aquifer matrix defined by the degree of karstification. The flow between SCX and WLD had lower groundwater velocity with more severe trailing of the breakthrough curve compared to LW to WLD, highlighting the varying degree of karst development. Consequently, the flow path from SCX to WLD was determined to be less porous, thereby retarding flow and solute transport.

Overall, the stronger the rainfall intensity, the larger the recharge volume driving stronger hydrodynamic conditions within the conduit. These conditions collectively generate higher groundwater velocity with advection playing a dominant role in the solute transport process, producing more symmetry and fewer tails on the breakthrough curve. Therefore, the period it takes the tracer to infiltrate into the aquifer matrix (fissures) becomes shorter, making the breakthrough curve exhibit a benign trailing phenomenon.

### 4.2. Simulations of Solute Transport Processes

Results of the tracer tests defined two major transport and storage paths: the linear pathway (mainly through conduits) and dynamic pathway (through fissures).

The solute transport process through conduits can be defined by a one-dimensional advection–dispersion equation. The conduit flow is characterized by high groundwater velocity, leading to a short travel time by the solute transport in the conduits with generally high concentration. The breakthrough curve shows a relatively thin and narrow shape (i.e., Figure 4a,c and Figure 5a,c). Obviously, the solute in the conduit flow is the first to reach the outlet of spring, and the rising process of the observed breakthrough curve basically reflects the arrival of the solute in the conduit flow.

Conversely, solute transport through fissures is characterized by low groundwater velocity and longer travel time associated with the storage phenomenon before releasing again to the conduits (Figure 2c). Furthermore, the mass of solute that can enter the fissure at high water level is limited due to the small spatial geometry of the fissure, making the peak concentration low.

The advection–dispersion equation is first used to fit the solute transport process of the linear transport path, which showed a good fitting effect under different hydrodynamic conditions, especially the rising process of the breakthrough curves (Figure 3a, Figure 4a,c and Figure 5a,c). This indicates that the first solutes reaching the spring outlet mostly come from the conduit flow. Consequently, the best advection–dispersion fitting curves can represent the solute transport processes in the conduit flow whose solutes only travel in the linear path. Regarding the dynamic pathway, it can be obtained by segmenting the fitting curves from the total breakthrough curves (Figure 3b, Figure 4b,d and Figure 5b,d), which are also the filled envelope areas in Figure 3a, Figure 4a,c and Figure 5a,c.

The calculated solute transport process still fit the advection–dispersion equation, especially in its rising stage before the peak concentration (e.g., Figure 4d and Figure 5b). However, small tails can be found in the estimated breakthrough curves for the dynamic pathway (e.g., Figure 4b and Figure 5d), indicating that there may be smaller fissures or pores that block solute transport. In the flow paths of SCX–WLD, with a lower mean discharge that indicated a smaller internal size and relatively lower karstification degree (Table 1), the linear pathway had a lower flow velocity, leading to more time for solute transportation into the dynamic pathway with higher tracer concentrations and longer duration times than that of LW–WLD (Figure 5).

### 4.3. Estimations of Solute Storage and Release

By integrating the envelope area of the total breakthrough curve and the simulated curve for the solute transport in the conduit flow, the total volume in the dynamic pathway can be estimated, which constitutes the mass of solute storage and release in the karst water system.

With increased recharge rate, hydrodynamic conditions increase while the solute storage decreases. On 12 July 2014, the rainfall intensity was the largest among these tracer test groups, leading to the strongest hydrodynamic conditions. The solute storage mass in the flow path of LW to WLD was 32.89 g, and that of SCX to WLD was 61.12 g (Table 1), while on 12 August 2014, rainfall intensity was smaller with less recharge rate, leading to higher values of solute storage mass in the two flow paths that were 67.53 g and 286.09 g, respectively. The tracer test of LJB to BLQ was conducted in the rainless condition with the weakest hydrodynamic condition, and its solute storage mass reached up to 1573.31 g with a highest storage rate of 26.22% among all the groups, while the storage rates of other groups conducted with higher rainfall intensity were no more than 10% (Table 1). Generally, in stronger hydrodynamic conditions or well-developed conduits (e.g., LW–WLD), the rates of solute storage in recovery are around 20%, while it can be over 40% in weaker hydrodynamic conditions (e.g., LJB–BLQ).

Under strong hydrodynamic conditions, fast flow from a single tracer injection point (sinkhole) can separate to a multiple flow path in the highly karstified area, resulting in not only a low recovery rate, but also a low storage rate. Therefore, with the increase in recharge rate through the sinkhole, the storage rate of tracers in a single conduit becomes lower. The reasons can be attributed to two aspects. First, the increase in hydrodynamic conditions leads to flow diverging to several conduits, which leads to the decrease in tracers flowing into a certain karst conduit. Second, under the condition of strong hydrodynamic force, the fast flow is concentrated and moves rapidly in the conduit with high velocity. The solute transport mainly performs in rapid advection, leading to the reduction of exchange time with the fissures, which reduces the solute storage in the fissures.

In the karst water systems of South China, due to the high heterogeneity in the development of subsurface conduits and fissures, the groundwater flows in different media are inconsistent. Some areas can form retention space such as the fissures, holes, and puddles that connect the conduits. When the tracer is temporarily stored in the retention area, the groundwater velocity decreases, and the advection function of solute transport becomes weaker, which is replaced by the mechanical dispersion function. When the stored solutes in the retention area are released into the conduits again, this part of solute will continue to mix with the conduit flow. Consequently, these stored solutes in the dynamic pathway produce a hysteresis effect, performing a trailing phenomenon in the breakthrough curves at the outlet (spring). In other words, the estimation of solute storage and release is the quantitative evaluation of the trailing phenomenon in the breakthrough curve.

For pollutant transport under concentrated recharge conditions, especially the sudden pollution incidents in karst depressions, the trailing phenomenon of pollutant transport determines the velocity and concentration distribution of the pollutant attenuation. Assessing the delay in the release of pollutant quantity and its temporal distribution has great significance in the prevention and control of karst groundwater pollution.

## 5. Conclusions

The current study validates the advection–dispersion method for the estimation of solute storage and release in karst water systems characterized by flow through conduits and fissures under autogenic recharge conditions.

Inter-exchange relationships between karst conduits and fissures were identified during different hydrodynamic conditions. Flow in the conduit travels much fast than that in the fissure, and there is a larger volume to store water in the conduit. When the water level in the conduit is higher than that of the fissure, flow, and its solute infiltrate into the fissure, they will come out again when the conduit water level becomes lower than that of the fissure.

Under autogenic recharge condition, solute transport was generalized into two flow paths (linear and dynamic). The largest mass transport occurs in the linear system characterized by large conduit flow directly from the sink (sinkhole) to the source (spring). The dynamic pathway is characterized by low groundwater velocity and longer travel time associated with the storage phenomenon before being released again to the conduits. The advection–dispersion equation was used to simulate the solute transport in a karst conduit, and a good fitness in the rising limb of breakthrough curves was obtained, which was also used to distinguish the solute transport in the two flow paths.

Based on five groups of artificial tracer tests conducted under different meteorological and hydrodynamic conditions, the mass of solute storage and release was calculated by segmenting the simulated curve in the linear pathway from the total tracer breakthrough curve. Under high flow conditions, the solute storage rates were generally less than 10% due to the large flow velocity and short residence time in the conduits. While the storage rate was up to 26.22% under low flow conditions due to its long trail in the breakthrough curve and longer exchange time for conduit flow and fissure flow. The flow velocity and residence time in the conduits are the main factors to control the inter-exchange of solute between karst conduits and fissures.

Overall, groundwater in karst areas with numerous depressions and sinkholes is highly susceptible to contamination due to autogenic recharge driven by overland flow. Runoff conveys contaminants from domestic waste, chemical input in agricultural activities, etc. This research highlights the solute transport mechanisms under these conditions and presents a method for estimating its volume to improve groundwater remediation and management.

## Figures and Tables

**Table 1:** Results of the artificial tracer tests and solute storage estimations.

Test Groups	LW–WLD 1	LW–WLD 2	SCX–WLD 1	SCX–WLD 2	LJB–BLQ
Hydrodynamic conditions	Higher level	High level	Higher level	High level	Low level
Tracer test date	12 July 2014	12 August 2014	12 July 2014	12 August 2014	5 July 2013
Injected tracer types	Uranine	Uranine	Rhodamine	Rhodamine	Rhodamine
Injected tracer mass (kg)	3	3	4	3	6
First detection time (h)	5.17	6.50	8.80	15.15	57.31
Mean discharge (L/s)	2821	2170	2146	1147	149
Max flow velocity (m/h)	976	776	413	240	56
Max concentration 1st (ppb)	11.77	40.24	12.72	31.58	19.25
Peak transit time 1st (h)	6.00	7.47	11.13	17.38	212.67
Mean transit velocity 1st (m/h)	841	676	327	209	15
Max concentration 2nd (ppb)	1.55	8.54	3.11	12.46	4.81
Peak transit time 2nd (h)	6.64	8.28	13.74	19.75	670.56
Mean transit velocity 2nd (m/h)	760	609	265	184	5
Dispersion (m^2^/s)	5.44	1.16	1.32	0.27	0.17
Recovery rate (%)	5.13	13.39	6.88	20.61	63.80
Solute storage mass (g)	32.89	67.53	61.12	286.09	1573.31
Rate of storage in recovery (%)	21.37	16.81	22.21	46.27	41.10
Solute storage rate (%)	1.10	2.25	1.53	9.54	26.22

Note: Mean discharge is the average value during main tracer breakthrough; 1st is short for the linear pathway; 2nd is short for the dynamic pathway. Max flow velocity is calculated by the first detection time and linear distance between injection and detection points. Mean transit velocity is calculated by the peak transit time and linear distance. Rate of storage in recovery is calculated by the solute storage mass and recovery mass. Solute storage rate is calculated by the storage mass and injected tracer mass. LW-WLD—Longwan-Wulongdong; SCX-WLD—Shicaoxi-Wulongdong; LJB-BLQ—Liujiaba-Bailongquan.
